# Chloroquine enhances TRAIL-mediated apoptosis through up-regulation of DR5 by stabilization of mRNA and protein in cancer cells

**DOI:** 10.1038/srep22921

**Published:** 2016-03-11

**Authors:** Eun Jung Park, Kyoung-jin Min, Kyeong Sook Choi, Peter Kubatka, Peter Kruzliak, Dong Eun Kim, Taeg Kyu Kwon

**Affiliations:** 1 Department of Immunology, Keimyung University, 2800 Dalgubeoldaero, Dalseo-Gu, Daegu 704-701, South Korea; 2 Department of Biochemistry & Molecular Biology, Ajou University School of Medicine, Suwon, South Korea; 3 Department of Medical Biology, Jessenius Faculty of Medicine, Comenius University in Bratislava, Martin, Slovakia; 4 Laboratory of Structural Biology and Proteomics, Faculty of Pharmacy, University of Veterinary and Pharmaceutical Sciences, Brno, Czech Republic; 5 Department of Pharmacology and Toxicology, Faculty of Pharmacy, Comenius University, Bratislava, Slovak Republic; 6 Department of Otolaryngology, School of Medicine, Keimyung University, 2800 Dalgubeoldaero, Dalseo-Gu, Daegu 704-701, South Korea

## Abstract

Chloroquine (CQ), an anti-malarial drug, has immune-modulating activity and lysosomotropic activity. In this study, we investigated CQ sensitizes TRAIL-mediated apoptosis in human renal cancer Caki cells. Combination of CQ and TRAIL significantly induces apoptosis in human renal cancer Caki cells and various human cancer cells, but not in normal mouse kidney cells (TMCK-1) and human mesangial cells (MC). CQ up-regulates DR5 mRNA and protein expression in a dose- and time- dependent manner. Interestingly, CQ regulates DR5 expression through the increased stability in the mRNA and protein of DR5, rather than through the increased transcriptional activity of DR5. Moreover, we found that CQ decreased the expression of Cbl, an E3 ligase of DR5, and knock-down of Cbl markedly enhanced DR5 up-regulation. Other lysosomal inhibitors, including monensin and nigericin, also up-regulated DR5 and sensitized TRAIL-mediated apoptosis. Therefore, this study demonstrates that lysosomal inhibition by CQ may sensitize TRAIL-mediated apoptosis in human renal cancer Caki cells via DR5 up-regulation.

Tumor necrosis factor-related apoptosis-inducing ligand (TRAIL), is a member of tumor necrosis factor gene superfamily, triggers apoptosis in a variety of cancer cell lines through binding of TRAIL to death receptors, and death-inducing signaling complex (DISC) is formed by following recruitment of Fas-Associated protein with Death Domain (FADD) and initiator caspases^1^,^2^. Interestingly, cancer cells are more sensitive to TRAIL than normal cells. Therefore, TRAIL received highly attention as a cancer therapeutic agent^3^,^4^. However, many cancers including renal cancer displayed TRAIL resistance, which is related with high expression levels of decoy receptors and anti-apoptotic proteins, mutation in TRAIL receptors, and dysregulation of DISC formation^5^,^6^,^7^. Therefore, for the successful development of TRAIL-based cancer therapy, identification of the effective sensitizer of TRAIL is required.

Chloroquine (CQ) is widely used as anti-malarial, anti-rheumatoid drug, and it is also reported as immune-modulating agent^8^,^9^. CQ has been reported for its potential use as a sensitizing agent in cancer therapies, and which is mainly through cell cycle arrest and growth inhibition in many cancers^10^,^11^,^12^. Also, molecular mechanism of the anti-cancer activity of CQ may be associated with inhibition of Akt signaling pathway^12^,^13^, activation of p53 pathway^14^, and inhibiting Bcl-2 Homology 3 (BH3) domain-mediated PUMA degradation^15^. Moreover, CQ can overcome drug resistance through sensitization of the chemotherapeutic agents, including BH3-mimetic ABT-737, anti-cancer agent 5-fluorouracil (5-FU) and PI3K/mTOR inhibitor PI103^16^,^17^,^18^,^19^ and radiation^20^,^21^,^22^ in several cancer cell lines. In addition, although CQ was shown to stimulate TRAIL-induced apoptosis in ABL-expressing HT29 cells, the molecular basis by which CQ sensitizes TRAIL-mediated apoptosis has not been fully investigated yet.

In the present study, we show that CQ sensitizes TRAIL-mediated apoptosis via up-regulation of DR5 through the stabilization of its mRNA and protein. These results provide the evidence that combined treatment with CQ may be a novel therapeutic approach for the successful TRAIL-based cancer therapy.

## Materials and Methods

### Cells and materials

Caki, MDA-MB-361 and U251MG cells were obtained from the American Type Culture Collection (ATCC) (Rockville, Maryland, USA). The culture medium used throughout these experiments was Dulbecco’s modified Eagle’s medium (DMEM) containing 10% fetal bovine serum (FBS), 20 mM HEPES buffer, and 100 mg/ml gentamycin. The mouse kidney cells, TMCK-1, were a gift from Dr T.J. Lee (Yeungnam University, Korea). Primary cultures of human mesangial cells (Cryo NHMC) and their corresponding growth medium (CC-3146 MsGM) were purchased from Clonetics (San Diego, California, USA). Recombinant human TRAIL and z-VAD-fmk were purchased from R&D system. CQ was purchased from Sigma Chemical Co. (St Louis, Missouri, USA). Anti-Bcl-2 (sc-783), anti-Bcl-xL (sc-634), anti-Mcl-1(sc-819), anti-cIAP2 (sc-7944), and anti-Cbl (sc-170) were purchased from Santa Cruz Biotechnology (Santa Cruz, California, USA). Anti-XIAP (610762) was purchased from BD Biosciences (Bedford, MA). Anti-pro-caspase-3 (ADI-AAP-113) antibody was obtained from Enzo life science (Farmington, NY). Anti-PARP (#9542) antibody, anti-cleaved caspase-3 (#9661S), anti-DR5 (#8074S), anti-cIAP1 (#4952S), anti-PSMA5 (#2457), and anti-PSMD/S5a (#1244) antibody were obtained from Cell Signaling Technology (Beverly, MA). Anti-actin (A5441) antibody was obtained from Sigma (St. Louis, MO). Other reagents were purchased from Sigma Chemical Co.

### Flow cytometry analysis

For flow cytometry, the cells were resuspended in 100 μl of phosphate-buffered saline (PBS), and 200 μl of 95% ethanol was added while the cells were being vortexed. The cells were then incubated at 4 °C for 1 h, washed with PBS, resuspended in 250 μl of 1.12% sodium citrate buffer (pH 8.4) with 12.5 μg of RNase and incubated for an additional 30 min at 37 °C. The cellular DNA was then stained by adding 250 μl of a propidium iodide solution (50 μg/ml) to the cells for 30 min at room temperature. The stained cells were analyzed by fluorescent-activated cell sorting on a FACScan flow cytometer to determine the relative DNA content, which was based on the red fluorescence intensity.

### Western blot analysis

For the Western blotting experiments, the cells were washed with cold PBS and lysed on ice in modified RIPA buffer (50 mM Tris-HCl pH 7.4, 1% NP-40, 0.25% Na-deoxycholate, 150 mM NaCl, 1 mM Na_3_VO_4_, and 1 mM NaF) containing protease inhibitors (100 μM phenylmethylsulfonyl fluoride, 10 μg/ml leupeptin, 10 μg/ml pepstatin, and 2 mM EDTA). The lysates were centrifuged at 10,000 × *g* for 10 min at 4 °C, and the supernatant fractions were collected. The proteins were separated by SDS-PAGE electrophoresis and transferred to Immobilon-P membranes. The specific proteins were detected using an enhanced chemiluminescence (ECL) Western blotting kit according to the manufacturer’s instructions.

### 4′,6′-Diamidino-2-phenylindole staining (DAPI) for nuclei condensation and fragmentation

To examine cellular nuclei, the cells were fixed with 1% paraformaldehyde on glass slides for 30 min at room temperature. After the fixation, the cells were washed with PBS and a 300 nM 4′,6′-diamidino-2-phenylindole solution (Roche, Mannheim, Germany) was added to the fixed cells for 5 min. After the nuclei were stained, the cells were examined by fluorescence microscopy.

### DNA Fragmentation Assay

DNA fragmentation was performed using the Cell Death Detection ELISA^PLUS^ kit (Boehringer Mannheim; Indianapolis, USA). Briefly, cells were centrifuged for 10 min at 200 × g, the supernatant was removed, and pellet was lysed for 30 min. After centrifuging the plate again at 200 × g for 10 min, and the supernatant that contained the cytoplasmic histone-associated DNA fragments was collected and incubated with an immobilized anti-histone antibody. The reaction products were incubated with a peroxidase substrate for 5 min and measured by spectrophotometry at 405 nm and 490 nm (reference wavelength) with a microplate reader. The signals in the wells containing the substrate alone were subtracted as background.

### Asp-Glu-Val-Asp-ase (DEVDase) Activity Assay

To evaluate DEVDase activity, cell lysates were prepared after their respective treatments with TRAIL in the presence or absence of CQ. Assays were performed in 96-well microtiter plates by incubating 20 μg of cell lysates in 100 μl of reaction buffer (1% NP-40, 20 mM Tris-HCl, pH 7.5, 137 mM NaCl, 10% glycerol) containing a caspase substrate [Asp-Glu-Val-Asp-chromophore-p-nitroanilide (DVAD-pNA)] at 200 μM. Lysates were incubated at 37 °C for 2 h. Thereafter, the absorbance at 405 nm was measured with a spectrophotometer.

### Determination of synergy and cell viability assay

The possible synergistic effect of CQ and TRAIL was evaluated using the isobologram method. In brief, the cells were treated with different concentrations of CQ and TRAIL alone or in combination. After 24 h, XTT assay was employed to measure the cell viability using WelCount Cell Viability Assay Kit (WelGENE, Daegu, Korea). In brief, reagent was added to each well and was then measured with a multi-well plate reader (at 450 nm/690 nm). Relative survival was assessed and the concentration effect curves were used to determine the IC_50_ (the half-maximal inhibitory concentration) values for each drug alone and in combination with a fixed concentration of the second agent^23^.

### RNA isolation, reverse transcription polymerase chain reaction (RT-PCR) and quantitative real-time PCR (qPCR)

Total cellular RNA was extracted from cells using TRIzol reagent (Life Technologies, Gaithersburg, Maryland, USA). Complementary DNA was synthesized from 2 μg of total RNA using M-MLV reverse transcriptase (Promega, Madison, WI, USA). The cDNA for DR5 and actin were amplified by a PCR using specific primers. The cDNA for DR5 was amplified by PCR with specific primers: DR5 (sense) 5′-AAGACCCTTGTGCTCGTTGT-3′ and (antisense) 5′-GACACATTCGATGTCACTCCA-3′. PCR products were analyzed by agarose gel electrophoresis and visualized using ethidium bromide staining. For quantitative real-time PCR, cDNA and specific primer pairs were mixed with SYBR GREEN Premix (TOYOBO, Japan), and performed on the LightCycler^®^ 480 real-time PCR system (Roche Diagnostics). The following primers were used for the amplification of DR5: DR5 (sense) 5′-AGACCCTTGTGCTCGTTGTC-3′ and (antisense) 5′-TTGTTGGGTGATCAGAGCAG-3′,. Threshold cycle number (Ct) of gene was calculated, and actin was used as reference genes. Delta–delta Ct values of genes were presented as relative fold induction.

### Transfection and Promoter Activity Assay

Cells were plated at approximately 60–80% confluence. The DR5 (Sac I) and DR5 (-605) promoter-constructs were transfected into the cells using Lipofectamine^TM^ 2000 (Invitrogen; Carlsbad, CA, USA). To assess the promoter-driven expression of the luciferase gene, the cells were collected and disrupted, and aliquots of the supernatants were used to analyze the luciferase activity according to the manufacturer’s instructions (Promega).

### Proteasome activity assay

Chymotryptic proteasome activities were measured with Suc-LLVY-AMC (chymotryptic substrate, Biomol International, Plymouth Meeting, PA). Lysate from CQ-treated cells was prepared. A mixture containing 1 μg cell lysate protein in 100 mM Tris-HCl (pH 8.0), 10 mM MgCl_2_, and 2 mM ATP was incubated at 37 °C for 30 min with 50 μM Suc-LLVY-AMC. Enzyme activity was measured with a fluorometric plate reader at an excitation wavelength of 380 nm and an emission wavelength of 440 nm. Also, fluorescence-based proteasome activity was assessed with ZsGreen (proteasome sensor vector) stably transfected Caki cell lines. Relative fluorescence activity was analyzed by using a FACS CantoTM (BD Biosciences, San Jose, CA, USA).

### Measurement of reactive oxygen species (ROS)

Intracellular accumulation of ROS was determined using the fluorescent probes 2′,7′-dichlorodihydrofluorescein diacetate (H_2_DCFDA). H_2_DCFDA is commonly used to measure ROS generation. Caki cells were pretreated with NAC and GEE for 30 min, and then added with CQ. Cells were stained with the fluorescent dye H_2_DCFDA for an additional 10 min. Then, cells were observed using a fluorescence microscope (Axiovert 200 M; Carl Zeiss).

### Analysis of cell surface DR5

Cells were detached with 0.5 mM EDTA, and washed three times with PBS. Washed cells were suspended in 200 μl of PBS, add primary antibody and incubated for 1 h at room temperature. Then, the cells washed twice with PBS, re-suspended in 200 μl of PBS and incubated with fluorescein isothiocyanate (FITC) conjugated secondary antibody for 30 min at room temperature. Unbounded secondary antibody was removed by centrifugation and cells were re-suspended in 500 μl of PBS. Cell-surface expression of DR5 was determined by flow cytometry.

### Small interfering RNAs

The GFP (control) and DR5 small interfering RNA (siRNA) duplexes used in this study were purchased from Santa Cruz Biotechnology (Santa Cruz, CA, USA). Cells were transfected with siRNA using Oligofectamine Reagent (Invitrogen, Carlsbad, California, USA) according to the manufacturer’s recommendations.

### Densitometry

The band intensities were scanned and quantified using the gel analysis plugin for the open source software ImageJ 1.46 (Imaging Processing and Analysis in Java; http://rsb.info.nih.gov/ij).

### Statistical analysis

The data were analyzed using a one-way ANOVA followed by post-hoc comparisons (Student–Newman–Keuls) using the Statistical Package for Social Sciences version 8.0 (SPSS Inc., Chicago, IL, USA).

## Results

### CQ enhances TRAIL-mediated apoptosis in human renal cancer Caki cells

**Figure 1:**
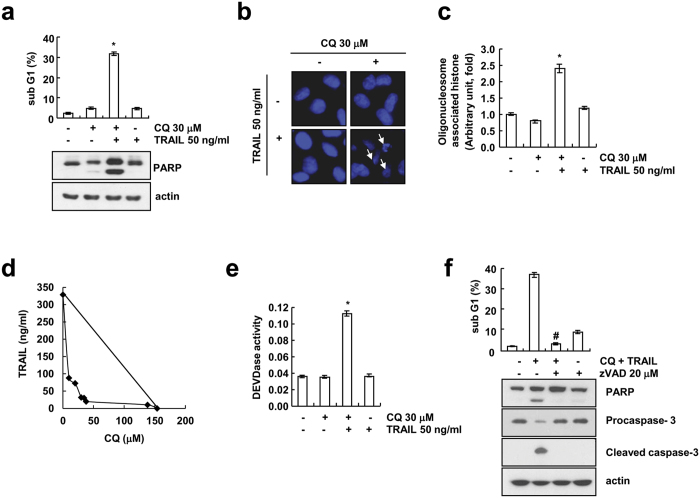
CQ sensitizes TRAIL-mediated apoptosis in human renal cancer Caki cells. (**a–c**) Caki cells were treated with the 50 ng/ml TRAIL in the presence or absence of 30 μM chloroquine (CQ) for 24 h. Apoptosis was analyzed as the sub-G1 fraction by FACS analysis. The protein expression of PARP was determined by Western blotting. The level of actin was used as a loading control (**a**). The condensation and fragmentation of the nuclei were detected by 4′,6′-diamidino-2-phenylindole (DAPI) staining (**b**). DNA fragmentation was determined using a DNA fragmentation detection kit (**c**). (**d**) Isoboles were obtained by plotting the combined concentrations of each drug required to produce 50% cell death. The straight line connecting the IC50 values obtained for two agents when applied alone corresponds to an additivity of their independent effects. Values below this line indicate synergy, whereas values above this line indicate antagonism. (**e**) Caki cells were treated with the 50 ng/ml TRAIL in the presence or absence of 30 μM chloroquine (CQ) for 24 h. Enzymatic activities of DEVDase were determined by incubation of 20 μg of total protein with 200 μM chromogenic substrate (DEVD-pNA) in a 100 μl assay buffer for 2 h at 37 °C. The release of chromophore p-nitroanilide (pNA) was monitored spectrophotometrically (405 nm). (**f**) Caki cells were pre-treated with 20 μM z-VAD-fmk (zVAD) for 30 min before combined treatment with 30 μM CQ and 50 ng/ml TRAIL for 24 h. Apoptosis was analyzed as the sub-G1 fraction by FACS analysis. The protein expression of PARP, procaspase-3 and cleaved caspase-3 were determined by Western blotting. The level of actin was used as a loading control. The values in panel (**a,c,e,f**) represent the mean ± SD from three independent samples. *p < 0.01 compared to the control. ^#^*p* < 0.01 compared to the CQ plus TRAIL.

To test whether CQ sensitizes TRAIL-mediated apoptosis, we checked cell death inducing effect of CQ alone, TRAIL alone or CQ plus TRAIL in human renal cancer Caki cells. Combined treatment of Caki cells with CQ and TRAIL significantly induces accumulation of sub-G1 population and cleavage of PARP, both are well-known apoptotic marker (Fig. 1a). In addition, we found that DNA fragmentation was induced only by CQ plus TRAIL, but not by a single treatment, when we performed the fluorescence miscopy using DAPI (Fig. 1b) and assessment of cytoplasm-associated with DNA fragments (Fig. 1c). Next, we examined whether combined treatment with CQ and TRAIL have synergistic effects. CQ plus TRAIL markedly reduced cell viability in various concentrations of CQ and TRAIL. The isobologram analysis suggested that combined treatment with CQ and TRAIL have synergistic effects (Fig. 1d). Activation of caspase-3 is a typical hallmark of apoptosis, and is proceeds to chromatin condensation and DNA fragmentation. Therefore, we analyzed whether co-treatment with CQ and TRAIL stimulates caspase-3 (DEVDase) activity. As shown in Fig. 1e, CQ or TRAIL alone did not stimulate caspase-3 activation, but combined treatment with CQ and TRAIL strongly activates caspase-3. To investigate the functional involvement of caspases, we examined the effect of z-VAD-fmk (zVAD), a pan caspase inhibitor, on the cell death induced by CQ plus TRAIL. We found that z-VAD-fmk pretreatment effectively blocked the increase in sub-G1 population (Fig. 1f) and the cleavage of caspase-3 and PARP (Fig. 1f). Collectively, these results indicate that combined treatment with CQ and TRAIL induces caspase-dependent apoptosis in Caki cells.

### CQ plus TRAIL induces apoptosis in other cancer cells, but not in normal cells

**Figure 2:**
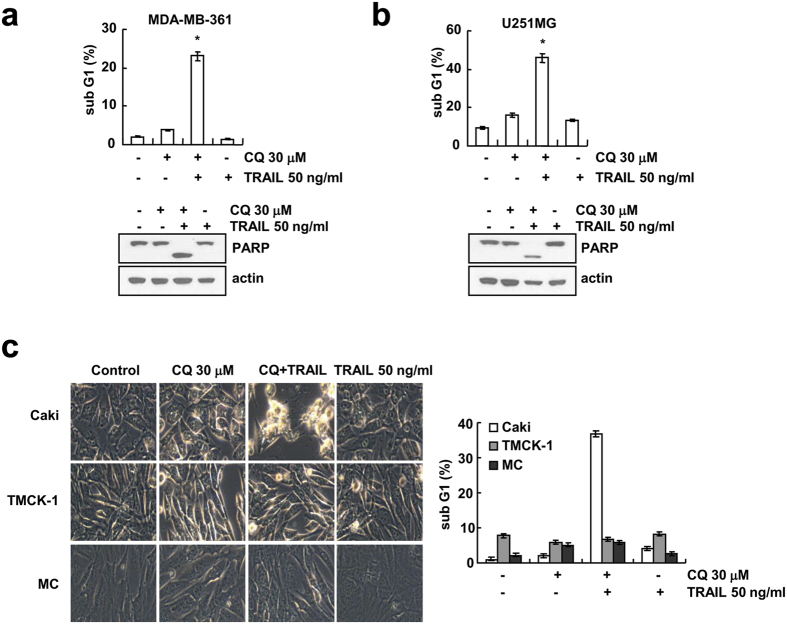
Effect of CQ plus TRAIL-induced apoptosis in other cancer cell lines and normal cells. (**a,b**) MDA-MB-361 and U251MG cells were treated with 50 ng/ml TRAIL in the absence or the presence of 30 μM CQ for 24 h. Apoptosis was analyzed as the sub-G1 fraction by FACS analysis. The protein expression of PARP was determined by Western blotting. The level of actin was used as a loading control. (**c**) Caki, TMCK-1 and MC cells were treated with 50 ng/ml TRAIL in the absence or the presence of 30 μM CQ for 24 h. The cell morphologies were determined by interference light microscopy. Images were magnified X200. Apoptosis was analyzed as the sub-G1 fraction by FACS analysis. The values in panel (**a–c**) represent the mean ± SD from three independent samples. *p < 0.01 compared to the control.

To investigate whether the sensitizing effect of CQ on TRAIL-mediated apoptosis is not restricted to a particular cell line, we examined the effect of CQ and/or TRAIL in other cancer cell lines. As shown in Fig. 2a,b, combined treatment with CQ and TRAIL markedly induced sub-G1 population and cleavage of PARP in MDA-MB-361 and U251MG cells. In contrast, combined treatment with CQ and TRAIL showed no morphological changes and did not increase sub-G1 population in TMCK-1 (mouse renal tubular epithelial cells) and MC (human mesangial cells) cells, differently from their effect in Caki cells (Fig. 2c). These results indicate that combined treatment with CQ and TRAIL might induce apoptosis in various cancer cells, but not in normal cells.

### CQ induces DR5 up-regulation in Caki cells

**Figure 3:**
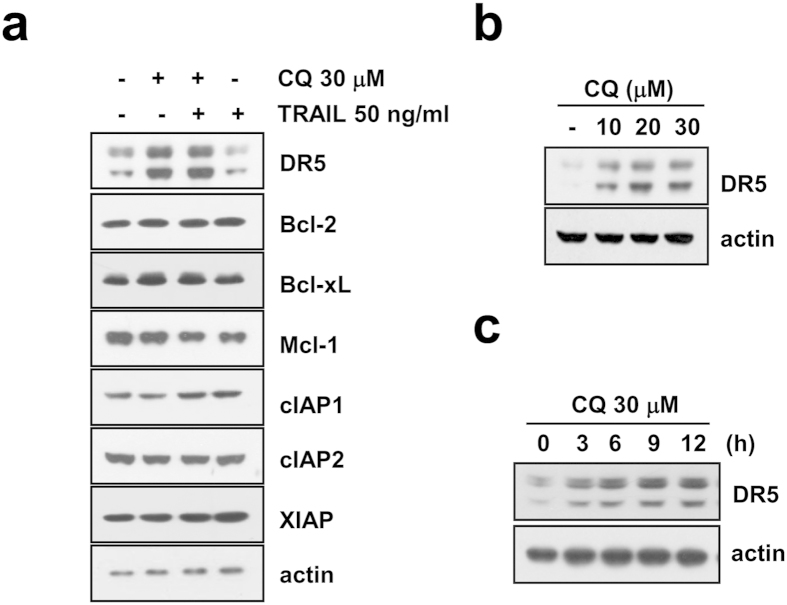
CQ induces DR5 up-regulation in Caki cells. (**a**) Caki cells were treated with 50 ng/ml TRAIL in the absence or the presence of 30 μM CQ for 24 h. The protein levels of DR5, Bcl-2, Bcl-xL, Mcl-1, cIAP1, cIAP2 and XIAP were determined by Western blotting. The level of actin was used as a loading control. (**b**) Caki cells were treated with indicated concentrations of CQ. The protein level of DR5 was determined by Western blotting. The level of actin was used as a loading control. (**c**) Caki cells were treated with 30 μM CQ for the indicated time periods. The protein level of DR5 was determined by Western blotting. The level of actin was used as a loading control.

**Figure 4:**
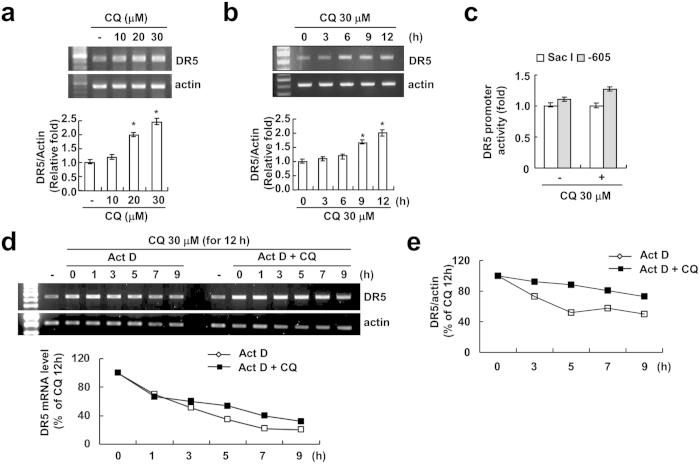
CQ induces DR5 up-regulation through mRNA stabilization in Caki cells. (**a**) Caki cells were treated with indicated concentrations of CQ for 24 h. The mRNA level of DR5 was determined by RT-PCR (upper panel) and qPCR (lower panel). The level of actin was used as a loading control. (**b**) Caki cells were treated with 30 μM CQ for the indicated time periods. The mRNA level of DR5 was determined by RT-PCR (upper panel) and qPCR (lower panel). The level of actin was used as a loading control. (**c**) Caki cells were transiently transfected with a plasmid harboring the luciferase gene under the control of the DR5/Sac I and DR5/-605 promoter. After transfection, the Caki cells were treated with 30 μM CQ for 24 h. After treatment, the cells were lysed, and the luciferase activity was analyzed. (**d,e**) Caki cells were treated 30 μM CQ for 12 h, then changed with fresh medium and pre-treated with 5 μg/ml actinomycin D (Act D) for 30 min, and treated with or without 30 μM CQ for the indicated time periods. The mRNA level of DR5 was determined by RT-PCR (**d**) and qPCR (**e**). The values in panel (**a–c**) represent the mean ± SD from three independent samples. *p < 0.05 compared to the control.

To explore the molecular mechanisms underlying CQ-mediated TRAIL sensitization, we first examined the protein levels of various apoptosis-related proteins. We found that the expression of Bcl-2, Bcl-xL, Mcl-1, cIAP1, cIAP2 and XIAP were not changed by CQ and/or TRAIL in Caki cells (Fig. 3a). In contrast, the protein levels of DR5, a TRAIL receptor, were markedly increased by CQ alone and CQ plus TRAIL. DR5 upregulation by CQ was observed in a dose- and time-dependent manner (Fig. 3b,c). CQ also induced DR5 expression in other cancer cells (Fig. S1). In contrast, CQ did not induce up-regulation of DR4 expression (Fig. S2). Next, we investigated whether CQ modulates DR5 expression at the transcriptional level. RT-PCR and qPCR analysis showed that CQ induced up-regulation of DR5 mRNA levels in dose- and time- dependent manner (Fig. 4a,b). To further confirm whether the DR5 upregulation by CQ treatment is through transcriptional activation, Caki cells were transfected with the plasmids with or without DR5 promoter (DR5/-605 or DR5/Sac I) and the change in the transcriptional activity of DR5 by CQ was examined. However, as shown in Fig. 4c, CQ did not noticeably induce the DR5 promoter activity. Therefore, we next investigated whether CQ regulates the DR5 mRNA stability. After treatment of Caki cells with CQ for 12 h, cells were treated with antinomycin D to stop *de novo* transcription in the presence or absence of CQ. When we measured mRNA degradation by RT-PCR and qPCR, CQ treatment attenuated the reduction of DR5 mRNA levels (Fig. 4d,e). These results indicate that the CQ-induced DR5 mRNA upregulation is associated with the enhancement of DR5 mRNA stability.

### CQ induces DR5 up-regulation through protein stabilization in Caki cells

**Figure 5:**
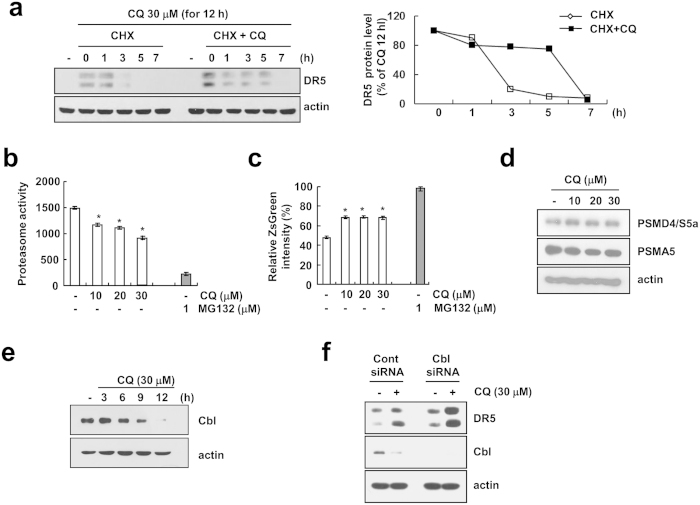
CQ induces DR5 up-regulation through sustained protein stability in Caki cells. (**a**) Caki cells were treated 30 μM CQ for 12 h, then changed with fresh medium and pre-treated 20 μg/ml cycloheximide (CHX) for 30 min, and treated with or without 30 μM CQ for the indicated time periods. The protein expression of DR5 was determined by Western blotting (left panel). The band intensity of DR5 protein was measured using the public domain JAVA image-processing program ImageJ (http://rsb.info.nih.gov/ij) (right panel). (**b**) Caki cells were treated with 1 μM MG132 (as a positive control) and indicated concentrations CQ for 24 h. The cells were lysed, and the proteasome activity was measured as described in the Materials and Methods. (**c**) Caki/ZsGreen cells were treated with 1 μM MG132 (as a positive control) and indicated concentrations of CQ for 24 h. Proteasome activity was analyzed by using FACS analysis. (**d**) Caki cells were treated with indicated concentrations of CQ for 24 h. The protein levels of PSMD4/S5a and PSMA5 were determined by Western blotting. (**e**) Caki cells were treated with 30 μM CQ for the indicated time periods. The protein levels of Cbl was determined by Western blotting. (**f**) Caki cells were transfected with Cbl siRNA or control siRNA. Twenty-four hours after transfection, cells were treated with 30 μM CQ for an additional 24 h. The protein expression of DR5 and Cbl were determined by Western blotting. The level of actin was used as a loading control. The values in panel (**b,c**) represent the mean ± SD from three independent samples. *p < 0.05 compared to the control.

To further examine the mechanism underlying CQ-mediated DR5 up-regulation, we investigated whether CQ modulates the protein stability of DR5 in Caki cells. After the treatment with CQ for 12 h, and cells were treated with cycloheximide (CHX), an inhibitor of *de novo* protein synthesis, in the presence or absence of CQ. CHX gradually decreased DR5 protein expression, but co-treatment with CHX and CQ more sustained DR5 protein expression (Fig. 5a). Next, we investigated whether proteasome is associated with CQ-mediated degradation of DR5. Analysis of both chymotryptic proteasome activity and fluorescence-based proteasome activity using ZsGreen (proteasome sensor vector) showed that CQ decreased proteasome activity (Fig. 5b,c). We examined expression levels of two critical proteasome subunits, 20S proteasome subunit type 5 (PSMA5) and 26S proteasome non-ATPase regulatory 4 (PSMD4/S5a), but CQ had no effect on expression of both subunits (Fig. 5d). When DR5 is degraded via the ubiquitin-proteasome pathways, ubiquitination of DR5 by Cbl E3 ligase is important^24^. We found that the protein expression of Cbl, one of the E3 ligases that targets DR5, was markedly decreased in a time-dependent manner in CQ-treated cells (Fig. 5e). Furthermore, siRNA-mediated knockdown of Cbl enhanced the up-regulation of DR5 induced by CQ (Fig. 5f). These data suggest that CQ induces up-regulation of DR5 expression at the post-translational level via down-regulation of Cbl expression.

### Reactive Oxygen Species (ROS) mediates CQ-induced DR5 up-regulation in Caki cells

**Figure 6:**
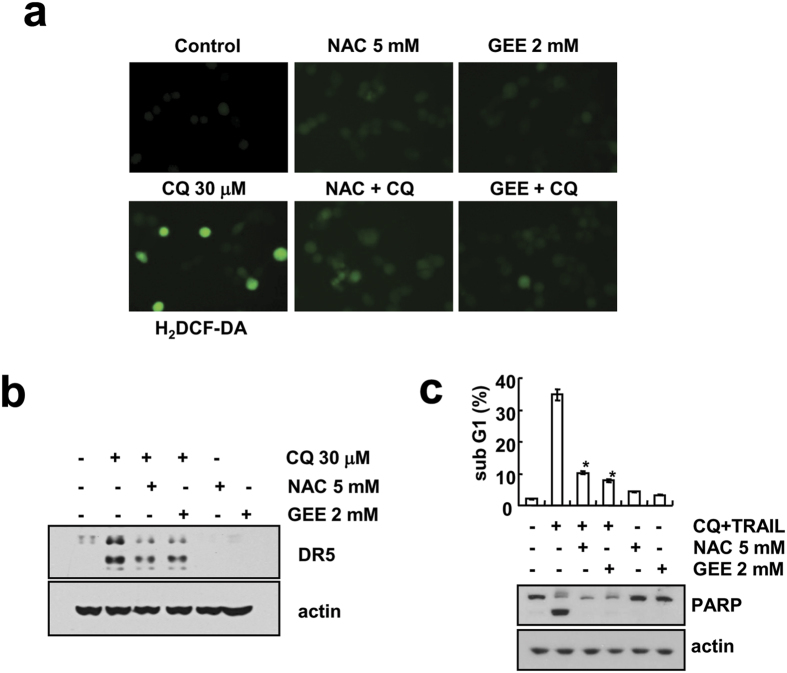
ROS generation is involved in CQ-induced DR5 up-regulation and CQ plus TRAIL-induced apoptosis in Caki cells. (**a**) Caki cells were treated with 30 μM CQ for 30 min in the presence or absence of 5 mM NAC or 2 mM GEE, and loaded with H_2_DCF-DA. H_2_DCF-DA fluorescence was analyzed by using a fluorescence microscopy. (**b**) Caki cells were treated with 30 μM CQ in the presence or absence of 5 mM NAC or 2 mM GEE. The protein expression of DR5 and actin were determined by Western blotting. The level of actin was used as a loading control. (**c**) Caki cells were treated with 30 μM CQ and 50 ng/ml TRAIL in the presence or absence of 5 mM NAC or 2 mM GEE. Apoptosis was analyzed as the sub-G1 population by FACS analysis. The protein expression of PARP and actin were determined by Western blotting. The level of actin was used as a loading control. *p < 0.01 compared to CQ plus TRAIL.

According to several reports, ROS generation is involved in DR5 up-regulation^25^,^26^. So, we checked involvement of ROS generation in CQ-induced DR5 up-regulation. H_2_DCF-DA-based ROS generation was analyzed by fluorescence microscopy, we observed increase of ROS generation in CQ-treated cells, and which was prevented by ROS scavengers [N-acetyl-L-cysteine (NAC) and glutathione ethyl ester (GEE)] (Fig. 6a). Also, pretreatment with NAC or GEE reduces the CQ-induced DR5 up-regulation (Fig. 6b). Because ROS generation is involved in CQ-induced DR5 up-regulation, its role in the CQ and TRAIL-induced apoptosis was further examined. As expected, CQ plus TRAIL-induced sub-G1 accumulation and PARP cleavage were reduced by ROS scavengers (Fig. 6c). These results indicate that DR5 up-regulation and TRAIL sensitize effect were dependent on CQ-induced ROS generation.

### DR5 up-regulation is associated with CQ plus TRAIL-induced apoptosis in Caki cells

**Figure 7:**
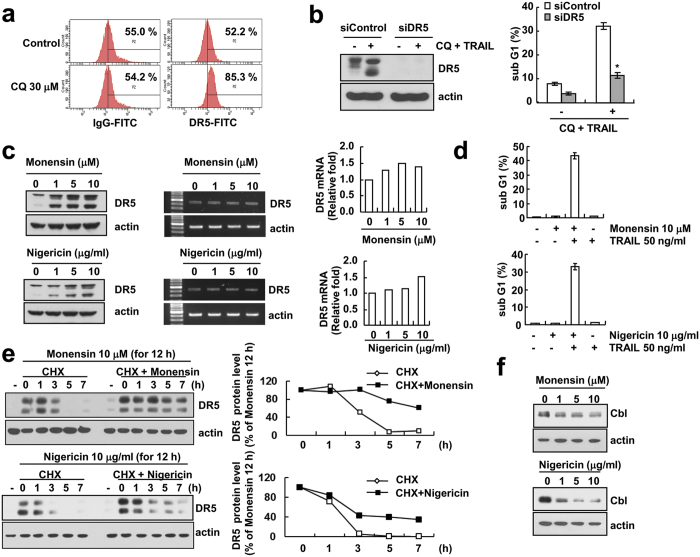
CQ-induced DR5 up-regulation is crucial for CQ plus TRAIL-induced apoptosis in Caki cells. (**a**) Caki cells were treated with 30 μM of CQ for 24 h. The cell surface expression level of DR5 was measured by flow cytometry analysis. (**b**) Caki cells were transfected with DR5 siRNA or control siRNA. Twenty-four hours after transfection, cells were treated with 30 μM CQ and 50 ng/ml TRAIL for an additional 24 h. The protein expression of DR5 was determined by Western blotting. Apoptosis was analyzed as the sub-G1 fraction by FACS analysis. (**c**) Caki cells were treated with indicated concentration of monensin or nigericin for 24 h. The protein level of DR5 was determined by Western blotting (left panel). The mRNA levels of DR5 was determined by RT-PCR(middle panel) and qPCR (right panel). (**d**) Caki cells were treated with the 50 ng/ml TRAIL in the presence or absence of 10 μM monensin or 10 μg/ml nigericin for 24 h. Apoptosis was analyzed as the sub-G1 fraction by FACS analysis. The protein expression of PARP was determined by western blotting. (**e**) Caki cells were treated 10 μM Monensin or 10 μg/ml Nigericin for 12 h, then changed with fresh medium and pre-treated 20 μg/ml cycloheximide (CHX) for 30 min, and treated with or without 10 μM Monensin or 10 μg/ml Nigericin for the indicated time periods. The protein expression of DR5 was determined by Western blotting (left panel). The band intensity of DR5 protein was measured using the public domain JAVA image-processing program ImageJ (http://rsb.info.nih.gov/ij) (right panel). (**f**) Caki cells were treated with 10 μM Monensin or 10 μg/ml Nigericin for 24 h. The protein expression of Cbl was determined by western blotting. The level of actin was used as a loading control. The values in panel (**b,d**) represent the mean ± SD from three independent samples. *p < 0.01 compared to the CQ plus TRAIL treated control siRNA.

Increased surface expression of DR5 is one of the essential points that implicated in TRAIL-mediated cell death. CQ enhances surface expression levels of DR5 (Fig. 7a). We also examined whether DR5 is key mediator of CQ plus TRAIL-induced apoptosis. The induction of apoptosis resulting from the combined treatment with CQ and TRAIL in Caki cells were effectively blocked by DR5 knockdown (Fig. 7b). Because CQ is lysosome inhibitor, we tested other lysosome inhibitors (monensin; Na^+^/H^+^ ionophore and nigericin; K^+^/H^+^ ionophore) also induce DR5 up-regulation and TRAIL-mediated apoptosis. As shown in Fig. 7c,d, monensin and nigericin induced DR5 up-regulation in Caki cells and sensitized TRAIL-mediated apoptosis. Interestingly, both drugs did not induce up-regulation of DR5 mRNA levels. Both lysosome inhibitors (monensin and nigericin) significantly enhanced DR5 protein stability compared to CHX alone (Fig. 7e). Furthermore, both lysosome inhibitors markedly induced down-regulation of Cbl expression in a dose-dependent manner (Fig. 7f). These results clearly showed that the lysosome inhibitors enhance DR5 expression at the post-translational level via down-regulation of Cbl expression.

## Discussion

In this study, we found the effect of CQ on TRAIL sensitization and the underlying molecular mechanism in renal cancer Caki cells. CQ up-regulates the DR5 mRNA and protein stability of DR5.

CQ has anti-cancer activity, including growth inhibition and induction of apoptosis. Mainly, CQ inhibits cell growth in leukemia, lung and colon cancer^10^,^11^,^13^, and induces G2/M arrest in breast cancer cells^12^. CQ also induced apoptosis through activation of p53 in glioma cells^14^ and inhibiting BH3 domain-mediated PUMA degradation in melanoma cells^15^. Moreover, CQ is used as sensitizer or enhancer in chemotherapy resistant cancer cells^16^,^17^,^18^,^19^. The sensitizer effect of CQ may be dependent or independent on autophagy inhibitory activity^17^. In addition, lysosome inhibitory activity of CQ has key roles in PI3K/mTOR inhibitor-resistant cancer cell death^19^.

CQ induces DR5 up-regulation through two different pathways. First, the up-regulation of DR5 mRNA expression is regulated by mRNA stability (Fig. 4c–e). As shown in Fig. 4d,e, treatment with CQ markedly enhanced half-life of DR5 mRNA. 3′-untranslated region (UTR) of DR5 contains several AU-rich regions capable of binding proteins such as HuR that post-transcriptionally regulate DR5 stability^27^. Down-regulation of HuR or ectopic expression of HuR had no effect on CQ induced up-regulation of DR5 mRNA (Negative data, Data not shown). These results indicate that the CQ-enhanced DR5 mRNA stability is not associated with HuR expression levels. We can’t rule out other poly (A)-binding proteins involved in 3′-UTR of DR5. Therefore, we need further investigation to identify the accurate mechanism of CQ-mediated DR5 mRNA stabilization. Second, up-regulation of DR5 protein is also mediated by protein stability, and it may be regulated by the inhibition of proteasome activity (Fig. 5b,c) and down-regulation of Cbl expression (DR5 E3 ligase). CQ reduces proteasome activity, and CQ caused a detectable increase in p62/SQSTM1^28^. Interestingly, CQ reduces proteasome activity in our system, but CQ did not regulate S5a and PSMA5, well-known proteasome subunits (Fig. 5d). Recently, Cbl has been shown to act as E3 ligase for DR5^24^. As shown in Fig. 5e,f, CQ treatment markedly decreased Cbl expression, and down-regulation of Cbl induced DR5 up-regulation. Our results demonstrated that CQ modulates DR5 expression via Cbl expression.

CQ is well known as a lysosomotropic agent^30^. Lysosomotropic agent has a lipophilic and basic moiety. Monensin and nigericin is a polyether antibiotic that acts as a carboxylic ionophore to bind Na^+^/H^+^ exchanger and K^+^/H^+^ ionophore, respectively^31^. The polyether ionophore antibiotics induce modification of permeability of cellular membranes to cationic metal species in lysosome^32^. Previous reports have shown that monensin enhanced TRAIL-mediated apoptosis via monensin-induced endoplasmic reticulum stress and CHOP-mediated DR5 up-regulation^33^. We examined whether other lysosome inhibitors showed similar activity on DR5 up-regulation. Both drugs did not induce up-regulation of DR5 mRNA level. We observed that monensin and nigericin also induced up-regulation of DR5, down-regulation of Cbl and sensitizes TRAIL-mediated apoptosis in Caki cells.

Taken together, our results clearly show that CQ-mediated up-regulation of DR5 effectively sensitizes to TRAIL-induced apoptosis in Caki cells, but not normal cells. These results may serve as novel therapeutic approaches of overcome TRAIL-resistant cancer therapy.

## Additional Information

**How to cite this article**: Park, E. J. *et al.* Chloroquine enhances TRAIL-mediated apoptosis through up-regulation of DR5 by stabilization of mRNA and protein in cancer cells. *Sci. Rep.* **6**, 22921; doi: 10.1038/srep22921 (2016).

## Supplementary Material

Supplementary Information
