# Supplementary material for: Chloroquine enhances TRAIL-mediated apoptosis through up-regulation of DR5 by stabilization of mRNA and protein in cancer cells
Source: Sci Rep. 2016 Mar 11;6:22921. doi: 10.1038/srep22921 (PMC4786792; doi:10.1038/srep22921)
Supplement: Supplementary Information [file srep22921-s1.pdf]

<Supplementary information>

Chloroquine enhances TRAIL-mediated apoptosis through up-regulation of DR5 by stabilization of mRNA and protein in cancer cells.

Eun Jung Park[1¶], Kyoung-jin Min[1¶], Kyeong Sook Choi[2], Peter Kubatka[3], Peter Kruzliak[4,5], Dong Eun Kim[6]*, Taeg Kyu Kwon[1]*

[1]Department of Immunology, Keimyung University, 2800 Dalgubeoldaero, Dalseo-Gu, Daegu 704-701, South Korea. [2]Department of Biochemistry & Molecular Biology, Ajou University School of Medicine, Suwon, South Korea. [3]Department of Medical Biology, Jessenius Faculty of Medicine, Comenius University in Bratislava, Martin, Slovakia. [4]2nd Department of Internal Medicine, Faculty of Medicine, Masaryk University, Brno, Czech Republic. [5]Department of Pharmacology and Toxicology, Faculty of Pharmacy, Comenius University, Bratislava, Slovak Republic. [6]Department of Otolaryngology, School of Medicine, Keimyung University, 2800 Dalgubeoldaero, Dalseo-Gu, Daegu 704-701, South Korea.

¶ These authors contributed equally to this work.

* Corresponding author: Taeg Kyu Kwon, Ph.D.
Address: Keimyung University, 2800 Dalgubeoldaero, Dalseo-Gu, Daegu 704-701, South Korea
Tel: 82-53-5803882
E-mail: kwontk@dsmc.or.kr

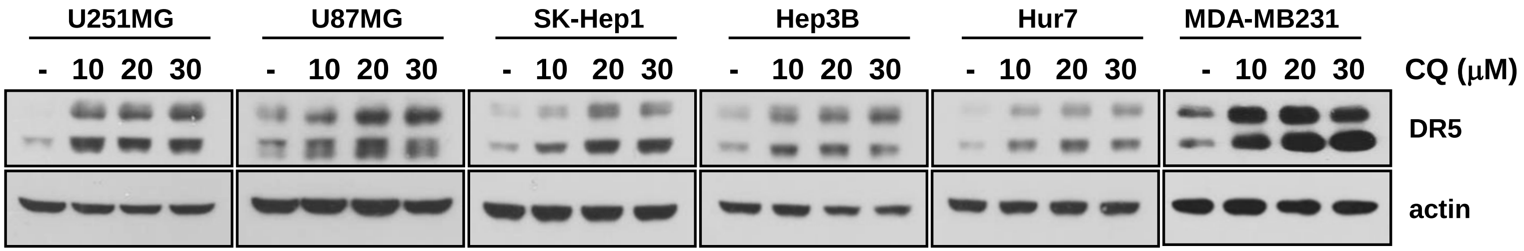

Figure S1. The effect of CQ on DR5 expression in other cancer cells. Human glioma (U251MG and U87MG), human hepatoma (SK-Hep1, Hep3B, and Hur7) and human breast cancer (MDA-MB231) cells were treated with indicated concentrations of CQ for 12 h. The protein level of DR5 and actin were determined by Western blotting. The level of actin was used as a loading control.

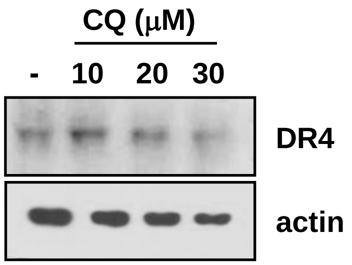

Figure S2. The effect of CQ on DR4 expression in Caki cells. Caki cells were treated with indicated concentrations of CQ. The protein level of DR4 was determined by Western blotting. The level of actin was used as a loading control.
